# Cognitive behavioral treatment for depressed adolescents: results from a cluster randomized controlled trial of a group course

**DOI:** 10.1186/s12888-019-2134-3

**Published:** 2019-05-22

**Authors:** Thormod Idsoe, Serap Keles, Asgeir Røyrhus Olseth, Terje Ogden

**Affiliations:** Norwegian Center for Child Behavioral Development, P.O. Box 7053, Majorstuen, 0306 Oslo, Norway

**Keywords:** Depression, Adolescents, Group-CBT, Randomized controlled trial

## Abstract

**Background:**

The group-based CBT intervention, *the Adolescent Coping with Depression Course (ACDC)*, has previously been evaluated within a quasi-experimental design, showing reduction in depressive symptoms compared to a benchmark of similar studies. The aim of our study was to investigate the effectiveness of ACDC within a randomized controlled (RCT) design.

**Method:**

Thirty-five course/control leaders randomly assigned to provide ACDC or usual care (UC) recruited 133 adolescents allocated to ACDC and 95 to UC. ACDC participants received eight weekly sessions and two follow-up sessions about 3 and 6 weeks after the last session. UC participants received usual care as implemented at the different sites. Depressive symptoms were measured with the Center for Epidemiologic Studies Depression Scale for adolescents (CES-D), perfectionism with the revised version of the Dysfunctional Attitude Scale (DAS), and rumination with the revised version of the Ruminative Responses Scale (RRS). Attrition was considered missing at random (MAR) and handled with a full information maximum likelihood (FIML) procedure.

**Results:**

Intention to treat analysis (ITT), including baseline scores and predictors of missing data as control or auxiliary variables, showed a small to medium reduction in depressive symptoms for the ACDC group compared to UC (d = −.31). Changes in perfectionism and rumination in favor of the intervention were also significant. Sensitivity analyses confirmed the findings from the ITT analyses.

**Conclusions:**

The current study supports the effectiveness of this group-based CBT intervention. The intervention can hopefully result in clinically significant reductions in symptoms associated with depression among adolescents.

**Trial registration:**

ISRCTN registry ISRCTN19700389. Registered 6 October 2015.

## Background

The *Adolescent Coping with Depression Course (ACDC)* is a group cognitive-behavioral program for depressed adolescents aged 14 to 20 years, with subclinical, mild or moderate depressive symptoms [1]. In 2015 and 2016, a cluster randomized controlled trial, in which the ACDC program was compared to usual care (UC) control, was implemented with the main aim of investigating the effects of ACDC on primary outcomes like depressive symptoms, and dropout among students in junior high school [2]. An additional aim was to investigate potential mediator/moderator variables such as dysfunctional attitudes, automatic thoughts, rumination and emotion regulation. While pre- and post-data for depressive symptoms and potential mediator/moderator variables have been gathered, the remaining primary outcome measures such as dropout will be collected from school registries after the follow-up studies. Consequently, the aim of this paper is to present the effects of ACDC on depressive symptoms (main primary outcome) and on the other variables mentioned above.

### Depression is a common mental health problem among adolescents

Depression is one of the most disabling diagnoses according to the World Health Organization [3], and one of the major contributors to the global burden of disease in terms of years of life lost to premature mortality [4]. Depression has its peak for first onset between the ages of 15 and 21 years [3, 5]. It is a serious problem for young people and one of the most common mental health issues for this age group [6]. Moreover, the number of adolescents being disabled by depression is increasing [7]. The prevalence among adolescents between 13 and 18 years is estimated to be 1–7% [8], indicating a considerable uncertainty related to the incidence estimates. The duration may vary, and depression has a high relapse rate, even after treatment [9]. Adolescent depression is associated with a range of problems, such as school dropout, school difficulties, health problems, increased substance abuse, as well as problems with peers and family [10–13], so it affects not only current, but also future functioning and health of the affected person. High quality early interventions could thereby be beneficial for student dropout rates as well as for academic motivation and social relations with peers.

Municipalities and mental health services in Norway have recently been strengthened when it comes to early identification, prevention and early treatment of illness [14]. Unfortunately, specialist mental health services still seem to reach few of these adolescents [8]. As many as 15–20% of Norwegian adolescents report considerable mental health problems [6], but only 16–17% of those have been in touch with mental health services about these issues [8, 15]. This suggests that a considerable number of adolescents in need of help do not receive it, and this may lead to challenges for schools in dealing with large groups of “unhelped” students. Failure to seek early treatment is associated with longer disease course and more relapsing episodes [16], and even subclinical depression among adolescents is associated with increased risk of depression and suicide attempts in adulthood [17]. Hence, providing treatment to young people may have a greater effect than treating adults. From a societal perspective, this means that even low-threshold interventions can be profitable. Taken together, this suggests that effective low-threshold interventions should be implemented. These interventions can be provided in places easily accessed by adolescents such as schools.

### Group-based cognitive behavioral therapy (CBT) interventions

Cognitive behavioral therapy (CBT) is one of the most effective interventions in treating depression among adolescents [18]. The main target in CBT is to modify and change maladaptive cognitions and behaviors that constitute the core processes of depression [19]. This is based on the hypothesis that behavior and emotions are influenced by people’s perception of events rather than the situation itself [20, 21]. Quick evaluative thoughts that we are barely aware of determine our perceptions, and depression is associated with having such automatic thoughts of a negative quality. Consequently, one important issue of CBT is to teach how to identify and modify such negative automatic thoughts.

Automatic thoughts are hypothesized to arise from more stable cognitions and coping styles that are thereby also hallmarks of depression [19]. Dysfunctional attitudes are one kind of such cognitive vulnerabilities. De Graaf, Roelofs, and Huibers [22] have demonstrated two sub-dimensions of this construct consisting of attitudes related to “dependency” and “perfectionism/performance evaluation”, both as risk factors for depression. While dependency consists of attitudes associated with the need for approval by others, perfectionism/performance evaluation consists of attitudes associated with fear of failure. Another cognitive style associated with depression is rumination which involves self-focused attention and a repetitive and passive focus on negative emotions [23].

Finally, a variable related to depression involves emotion regulation strategies. According to the cognitive model, painful negative emotion is related to misinterpretation of situations, and successful treatment should teach individuals how to reduce this emotional distress by enhancing positive ways of regulating emotions.

Group-based CBT interventions for depressed adolescents have proven effective for adolescents with diagnoses of major depression or dysthymia [24–27], but also when used to prevent further development of subclinical depression among adolescents [28, 29]. A recent meta-analysis of group-based CBT for adolescents in RCT design demonstrated a standardized mean difference of .28 compared to controls [30]. This d-score is comparable to that reported by Weisz, McCarty, & Valeri [31] (.34), and to the meta-analysis of the international “Coping with Depression” (CWD) course distributed to adolescents [32] (.35), but somewhat lower than reported by Klein, Jacobs, & Reinecke [33] (.59). One potential reason could be that Klein et al. [33] focused exclusively on RCT’s involving adolescents with depressive diagnoses, while the others included studies of youths with varying degrees of depressive symptomatology. We would thereby expect an effect comparable to Keles and Idsoe [30], Weisz et al. [31] and CWD [32].

ACDC may have many similarities with the “Coping with Depression Course” (CWD), developed by Lewinsohn and colleagues [25], but CWD has not been translated or tested in Norway. ACDC as developed in the Norwegian context also has new components, making a randomized controlled trial of this specific treatment as a contribution to the field. The new components include updated approaches and techniques from Rational Emotive Behavior Therapy (REBT) [34] and Cognitive Behavioral Therapy (CBT) [20]. Moreover, ACDC also includes elements from Meta-Cognitive Theory (MCT) [35],Positive Psychology (PP) [36] and modern neurobiological perspectives.

While CBT focuses on thinking style, MCT focuses on how to reflect on your thinking style within a meta-perspective, by being consciously aware of how you can “think about your own thoughts” [35]. In ACDC these perspectives are combined in order to help how to deal with the way thoughts can change in terms of quality and style, and hopefully improve the potential for modifying dysfunctional thoughts. This may be especially useful for rumination, which has been suggested as one of the drivers of depressed mood. Affect regulation is also another important component in the course.

PP is used for breaking thinking patterns, based on a neurobiological understanding of how thinking patterns, behaviors and emotions are formed and developed. Because PP is less documented as a single initiative for young people (like MCT), ACDC combines it with the other perspectives. For example, it emphasizes awareness and training in breaking thought patterns (in line with CBT) as one tool.

The neurobiological perspective is used to give adolescents a better understanding of how information is processed and why individuals react the way they do, sometimes automatically. Based on these conceptual understandings, the adolescents can root their work with the concrete techniques in a solid base.

Further, ACDC is trying to reach a broad range of difficulties because depressive mood often does not occur as the only difficulty, so a slightly broader ‘transdiagnostic’ initiative is desirable.

Because context is considered important for symptom relief, separate pamphlets are distributed to family/school/work place.

## The current study

The ACDC has previously been evaluated within a quasi-experimental design, giving a standardized mean reduction in symptoms of .45, compared to a benchmark of similar studies [37]. The aim of the current study was to investigate the effectiveness of ACDC within a randomized controlled trial. Our main outcome was depressive symptoms, but we also wanted to investigate effects on potential mediator/moderator variables such as negative automatic thoughts, dysfunctional attitudes, rumination and increased positive emotion regulation strategies.

## Method

### Recruitment and randomization of course leaders

Our design was a two-arm parallel cluster randomized controlled trial, with course leaders as the unit of allocation and youth participants as the unit of analysis. Cluster randomization by course leaders rather than by adolescents provided easier access to depressed young people through school and health systems and minimized the potential contamination between intervention and control groups.

Course leaders were recruited from the Educational Psychological Services, school mental health offices, the Children and Young People’s Psychiatric Out-patient Services.

Out-patient Services (BUP) over two consecutive school years. Certification as an ACDC course leader requires completion of a minimum 3–year college/university education (e.g. psychology, education, health, or related disciplines) before attending the 5-day certification course. 97% of the trainers were female and they were mostly counselors/special educators at school (44%), school/health nurses (26%), social workers (15%), psychologists (12%), and the rest had various roles such as family therapists or physiotherapists. All were employed in community health, public health/public hospitals, schools or in private healthcare. Fifty-eight course leaders were recruited to participate in the study via various channels such as mass mailing to upper secondary schools, school health services, educational follow-up services. These course leaders were randomized to experimental (k = 31) and control (k = 27) conditions by administrative personnel at the Norwegian Center for Child Behavioral Development. Course leaders for the intervention group received their training for a full week before the recruitment of course participants and the intervention periods started, while the leaders of “usual care” (UC) received a one-day training in how to recruit adolescents in order to standardize the recruitment process. The course leaders randomized to the control condition were offered the full training in the ACDC intervention shortly after they had finished conducting the control treatment groups (UC) so they could benefit from the training without affecting the study results.

**Fig. 1:**
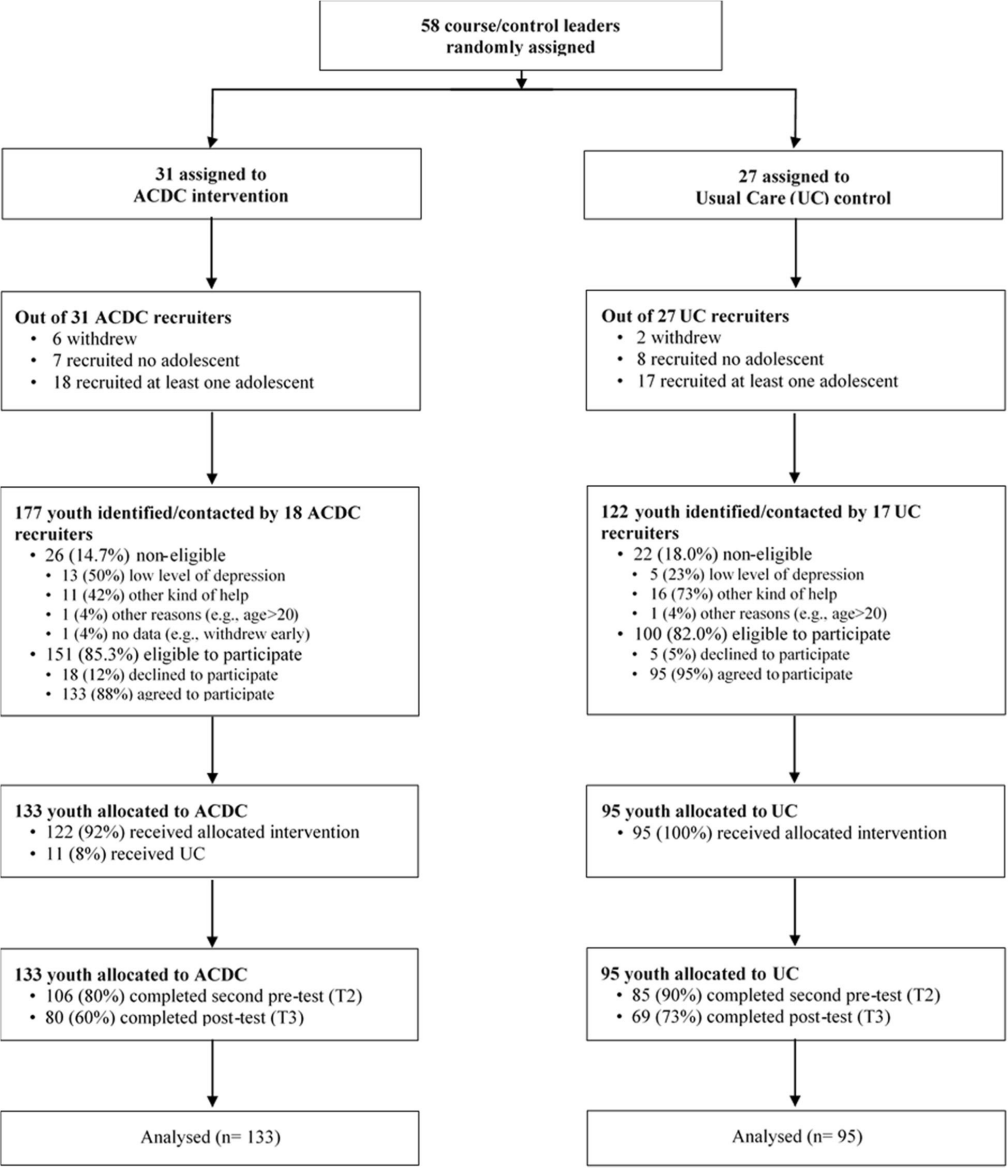
Participants flowchart

Of the 58 course leaders, 8 withdrew at the beginning of the study period, 15 of them recruited no participants so were not able to run ACDC or UC. Eventually, 18 course leaders randomized to experimental and 17 leaders randomized to control condition were included in the study (see Fig. 1).

#### Screening and inclusion of participants

Participants, adolescents with depressive symptoms, were recruited by the ACDC/UC leaders, through various channels including by placing information in schools and health centers, providing information through GPs, advertisements in local newspapers, on websites for young people, information at the local hospital’s medical meetings. Participants were recruited either by making direct contact themselves with course leaders, or they were referred by health visitors, GPs, psychiatric clinics, municipal services, school counselors, and the like. The target population was students from the 1st or 2nd grade of upper secondary school, who were 16 and 17 years old. There was a maximum cut-off age of 20 years and subjects had to have subclinical depression or mild to moderate depression, according to the criteria of the DSM. Exclusion criteria included presence of bipolar disorder, psychosis, substance-use, ADHD or ADD and brain damage as listed in the ACDC manual. Language abilities good enough to follow the course is required.

Potential study participants were screened for eligibility and provided with study information in a semi-structured interview conducted by the ACDC/UC leaders. To reduce post-randomization selection bias, it was important that the ACDC/UC leaders did not reveal what condition they were recruiting for. This means that the adolescents received information about both conditions and signed the consent for whichever intervention they would receive. At the first meeting with the course/control leaders, adolescents were screened using the Beck Depression Inventory (BDI) [38] and had the brief clinical interview. This interview was also the mechanism for determining whether the participants met criteria for the exclusionary diagnoses (e.g., ADHD). Adolescents who had a BDI score > 10 would satisfy the recommended cutoff criterion for mild to moderate depression [39]. If they in addition were between 16 and 20 years old, they were eligible to be included in the study. If they agreed to participate, written consent was obtained and the first pre-test of baseline measures was administered at the end of the interview. Due to difficulties in recruiting enough participants, we had to repeat the study 1 year after. Adolescents were thereby recruited in two cohorts, the first throughout November and December 2015 and the second throughout November and December in 2016. In total, 228 adolescents (88% girls; Mean_*Age*_ = 16.70 years, *SD* = 1.14) were recruited.

### Interventions

#### Intervention group: the “Adolescent Coping with Depression Course” (ACDC)

The “Adolescent Coping with Depression Course” (ACDC) is a CBT-based group course for adolescents with subclinical or mild to moderate depression developed for the Norwegian context [1]. The development was funded by the Norwegian Directorate of Health. As explained in the introduction, ACDC have many similarities with "the Coping with Depression Course (CWD), but the latter has not been translated or tested in Norway, and ACDC is also more developed by being based on the new and unique components that we described initially.

The importance of practicing the techniques learned throughout the course is emphasized both in and outside the course setting to develop the necessary skills. One of the goals of the course is for the adolescents to acquire a ‘toolbox’ of skills and techniques to help them cope better with their depressive symptoms in the future. The material includes a manual for the facilitator, a course pamphlet for the participants, a pamphlet addressed to parents and also a pamphlet for the school/workplace as well. Additionally, a short downloadable presentation has been developed for teachers to use in class to present mental health as a topic if required. All the course materials are printed and published by the Norwegian Council for Mental Health (NCMH).

The course is usually delivered in a group format over eight consecutive weekly sessions, each lasting approximately 120 min, with breaks. If necessary, it could be delivered twice a week over a shorter period. Two follow-up sessions are conducted about three and 6 weeks after the last session, lasting approximately 90 min. In total, the ACDC consists of 10 sessions. Ideally, the sessions are at the same time every week, and each session has a specified topic described above. Generally, the sessions start by summarizing the previous session and reviewing the homework assignments. An overview of the course is given in the Appendix. The location for where the ACDC groups were run depended on where the providers practiced.

#### Control group: usual care (UC)

The participants in the control condition received usual care, i.e. the treatment active control course leaders would typically employ for this group. This may involve referring them to very different care providers (psychologists, doctors, school nurses, teachers) who may provide, for example, conversations, various standard treatments, the use of pharmacotherapy or no treatment. No restrictions were put on what the young people in the control group could receive. The participants and UC leaders were asked to report who they referred/were referred to and who they received care from.

### Measures

In addition to the demographic variables including gender and age, the youth participants provided information on the following measures.

#### Depressive symptoms

The level of depressive symptoms was assessed by the Center for Epidemiologic Studies Depression Scale for adolescents (CES-D) [40]. CES-D asks for the frequency of symptoms during the last week of depressed affect (7 items), lack of positive affect (4 items), somatic and retarded activity (7 items), and interpersonal problems (2 items). A four-point Likert scale was used, with a total score ranging from 0 (*no symptoms*) to 60 (*high level of and frequent symptoms*). A previous confirmatory factor analysis supported the use of four dimensions invariantly to gender and differing ethnic backgrounds among adolescents in Norway [41]. CES-D has been extensively used to assess depressive symptoms in adolescents [42]. A conservative diagnostic cutoff of 28 or higher has been suggested for this age group based on DSM-criteria [43]. Cronbach’s alphas for the total symptom scale in the current study varied between .88 and .92 across waves.

#### Negative automatic thoughts

The frequency of negative thoughts was assessed using the single-factor, 8-item short-form version of the Automatic Thoughts Questionnaire (ATQ) [44, 45]. This short version of ATQ shows high reliability and validity, without deteriorating content domain coverage of the construct [45], and this has also been established among undergraduates [46]. The frequency of negative thoughts was rated on a 5-point scale ranging from 1 (*not at all*) to 5 (*all the time*). A sample item included “I’m so disappointed in myself”. In this study, the Cronbach’s alphas ranged between .88 and .93.

#### Dysfunctional attitudes

The intensity of dysfunctional attitudes was measured with the revised version of the Dysfunctional Attitude Scale (DAS) [22] which is one of the most commonly used instruments as a mediator of outcome in CBT for depression [47]. The scale has previously demonstrated good psychometric properties among adolescents [48]. Two-factor solution with 17 items, consisting of ‘dependency’ and ‘perfectionism/performance evaluation’ dimensions, demonstrated good reliability and convergent construct validity [22]. Sample items included “My value as a person depends greatly on what others think of me”, and “It is difficult to be happy, unless one is good looking, intelligent, rich and creative”, for ‘dependency’ and ‘perfectionism/performance evaluation’ dimensions respectively. Items were rated on a 7-point Likert scale ranging from 1 (*strongly agree*) to 7 (*strongly disagree*). Lower scores indicated more intense dysfunctional attitudes. In this study, the Cronbach’s alphas for the total scale ranged between .90 and .92.

#### Rumination

Revised version of the Ruminative Responses Scale (RRS) [23], a self-report measure of rumination with 10 items, was used to measure two aspects of rumination, ‘reflection’ and ‘brooding’. This version of RRS does not include items that seem to overlap with the measures of depressive symptoms [42]. Respondents rated each questionnaire item on a scale from 1 (*almost never*) to 4 (*almost always*). Sample items included “Write down what you are thinking and analyze it” for reflection, and “Think about a recent situation, wishing it had gone better” for brooding dimensions. This scale has demonstrated good psychometric abilities among adolescents in previous studies [49]. In this study, the Cronbach’s alphas for the total scale ranged between .78 and .86.

#### Emotion regulation

Emotion regulation strategies were assessed through the Norwegian version of the Emotion Regulation Questionnaire (ERQ) [41, 42]. The ERQ is a 10-item measure that assesses the use of two common emotion regulation strategies: cognitive reappraisal and expressive suppression. Sample items included “I control my emotions by changing the way I think about the situation I’m in” (reappraisal) and “I control my emotions by not expressing them” (suppression). Items were rated on a scale from 1 (*strongly disagree*) to 7 (*strongly agree*). It has been concluded that this is a valid age-appropriate measure for investigating cognitive reappraisal and expressive suppression during the childhood and adolescence developmental periods [50]. In this study, the Cronbach’s alphas for the total scale ranged between .67 and .74.

#### Course leaders’ retrospective evaluations

Both ACDC and UC leaders were asked to retrospectively evaluate whether they perceived the course/usual care delivered as effective for each of the adolescents separately. The item was rated on a scale from 1 (*No, things got worse*) to 4 (*yes, it helped a lot*). The course leaders also had a “do not know” option for their evaluations.

#### Course leaders’ self-reported fidelity

Adherence to core treatment components was measured at the conclusion of treatment by asking the course leaders of the intervention group to what extent they covered the six topics: *emotion regulation*, *training tasks at home*, *the abc model (understanding own reactions/emotions)*, *significance of own thoughts*, *challenging own thoughts*, *strengthening social relations*. The six items were rated on four ordinal categories: 1 (rarely or never), 2 (sometimes), 3 (often), 4 (mostly).

### Procedure

Data were collected via self-reported questionnaires. The first pre-test of baseline measures (T1) was administered at the end of the screening interviews as a paper-and-pencil format. The screening period lasted from November through December, resulting in individually varying times of the T1 assessments. The trial started in January 2016 for the first cohort and January 2017 for the second, and ran for 14 weeks, either by 10 sessions of the ACDC (treatment condition) or UC for the control condition. Two weeks before the trial, the main outcome measure of depression was assessed as the second pre-test (T2) via electronic questionnaire. Due to the individually varying times of T1 assessments, the period from T1 to T2 varied for the adolescents. After the intervention period ended, the participants received an electronic post-intervention questionnaire with all study measures (T3). The questionnaire took approximately 30 min to complete. Course leaders’ self-reported fidelity was measured at the conclusion of treatment.

### Sample size calculation

Since the design of this study is a cluster-randomized effectiveness trial with active control, where groups rather than individuals were randomized, possible cluster effects should be accounted for in calculating appropriate sample size. Data from the previous study on ACDC [37] was used to calculate intraclass correlations (ICC) for the main outcome, depressive symptoms, with the course groups as cluster. We used this approximation for our power calculations [51]. With a .05 level of significance, power = .80, ICC = .08, number of clusters = 25, cluster size = 8, *N* = 200, we would be able to detect effect sizes of .49. We recruited 35 course leaders (clusters) who recruited 228 adolescents, and the average cluster size was 6.5.

### Statistical analyses

The primary analyses were based on the principle of intention to treat (ITT) in accordance with the Consolidated Standards of Reporting Trials (CONSORT) [52, 53]. SPSS 21 was used for descriptive analyses, while Mplus 7.3 [54] was used for analyzing the repeated data by examining autoregressive models of latent variables at pre-test and post-test stages (observed variables for per protocol analyses because of sample size restrictions). Effect sizes for the intervention may be interpreted as a standardized mean difference. The robust ML (maximum likelihood) estimator was preferred to accommodate non-normal item distributions and missing data. We employed the full information maximum likelihood (FIML) procedure which uses all available data points [47] and is consistent with the ITT analysis approach. In addition to the chi-square statistic, which is sensitive to sample size [48] and seldom non-significant in large enough samples, other fit indices were additionally consulted: the root-mean-square error of approximation (RMSEA), the comparative fit index (CFI), the non-normed fit index (Tucker Lewis Index - TLI), and the standardized root-mean-square residual (SRMR). West, Taylor, Wu, and Hoyle [55] suggest that CFI > .95, RMSEA < .05, and SRMR < .06 represent a well-fitting model. They also further suggest that CFI > .90, RMSEA < .08, and SRMR < .10 represent an adequately fitting model.

Even though the unit of analysis was the youth participants, the course leaders were the unit of allocation. This cluster randomization may have caused potential bias in estimates of standard errors. To control for this, as the first step, we also calculated the intraclass coefficients (ICC) of our outcomes.

### Missing data

A careful and thorough approach to dropout and missing data is very important in intervention research [56, 57]. Steps were taken throughout the trial design and trial conduct to reduce dropout. In addition, the FIML approach as implemented in our analytical software Mplus can reduce potential bias due to missing data. These procedures are recommended in the leading modern methodological literature [58] as well as by the Panel on Handling Missing Data in Clinical Trials [57]. However, the application of these methods relies on assumptions about the factors leading to missingness and how they relate to the outcomes of interest. We considered data to be “missing at random” (MAR). It is very important to describe fully the arguments underlying these assumptions. Please see the Appendix for more details on these issues.

The National Research Council [57] suggests that sensitivity analyses should be conducted in order to illuminate the degree to which potential treatment effects rely on the assumptions used. We conducted two such sensitivity analyses to investigate how sensitive the intervention effects were to our MAR assumptions. First, we conducted per protocol analyses. Second, we conducted the analyses based on the ITT sample, but relaxing the MAR assumptions to “missing not at random” (MNAR) by removing auxiliary information from the analyses. Please see Appendix for more details.

## Results

### Participant flow

Because we had difficulties recruiting enough participants in the first cohort, we recruited a second cohort the following year so we got two cohorts (November/December 2015 and November/December 2016). In total, 35 course/control leaders randomly assigned to treatment condition were included in the study. For ACDC, a total of 177 adolescents were identified for the initial consultation by 18 ACDC course leaders. Thirteen of them were non-eligible due to low levels of depression. An additional 11 adolescents were referred to other kinds of help in accordance with exclusion criteria. One was excluded due to age, and one withdrew during the consultation interview. Altogether this means that 26 (14.7%) were non-eligible, while 151 (85.3%) were eligible to participate. Eighteen of the eligible adolescents refused to participate. Of the remaining 133 adolescents, 122 received the allocated ACDC. The rest (11 adolescents) received UC. The reason for this was that the course leaders did not manage to recruit enough participants to achieve minimum group size for ACDC. Of the 133 adolescents allocated to ACDC, 106 (80%) completed second pre-test (T2) and 80 (60%) completed the post-intervention test (T3).

For UC, 122 adolescents were identified for the initial interview and screening by 17 UC control leaders. Five of them were excluded due to low levels of depression, and 16 were referred to other kinds of help. One adolescent withdrew almost immediately. This means that 22 adolescents (18%) were non-eligible and 100 adolescents were eligible to participate. Of those, five declined to participate, resulting in a net sample of 95 eligible adolescents who were allocated to UC. Eighty-five of them (89.5%) completed the second pre-test (T2) and 69 (73%) completed the post-intervention test (T3).

Figure 1 shows the participant flowchart. The attrition group for post-test did not significantly differ from those who continued in regard to the study variables of interest or demographic variables (see Appendix Attrition). There was only one group difference for the second pre-test; that is, the attrition group for the second pre-test consisted of older participants compared to those who had second pre-test data, *t*(227) = 3.061, *p =* .002. The inclusion of age as auxiliary information for our ITT analyses handles this by use of the FIML procedure (Enders, 2010).

### Cluster effects

In accordance with CONSORT [52, 53], potential cluster effects must be accounted for in the analyses. Intraclass correlations (ICC’s) for our outcomes ranged from .012 to .099, indicating that more than 90% of the variance was at the individual (adolescent) level. However, whether the sizes of the ICC’s indicate salient cluster effects depends on the average cluster size. The design effect is a function of the intraclass correlation and the average cluster size that has to be evaluated. Conventionally, a design effect greater than 2 means that the clustering must be taken into account when estimating models [59]. The average cluster size in our data was 6.514, and none of the design effects were higher than 1.55. This indicates that the clustering will exert no practical influence for our analyses, hence the results of the analyses without correction for clustering is reported.

### Baseline characteristics

Descriptive statistics with the means and standard deviations for both conditions at pre-tests and post-test are portrayed in Table 1.

**Table 1:** Descriptive statistics by condition

	ACDC intervention (*N* = 133)						UC control (*N* = 95)					
	T1- Pre-test		T2- 2nd Pre-test		T3- Post-test		T1- Pre-test		T2- 2nd Pre-test		T3- Post-test	
	*M*	*SD*	*M*	*SD*	*M*	*SD*	*M*	*SD*	*M*	*SD*	*M*	*SD*
Depression	33.08	9.97	32.77	8.80	26.85	11.82	32.01	9.75	30.28	10.67	29.55	10.77
Negative automatic thoughts	3.05	0.89	–	–	2.66	1.04	2.98	0.98	–	–	2.79	1.04
Dysfunctional attitudes - *Perfectionism/Performance evaluation*	4.01	1.16	–	–	4.42	1.28	4.06	1.14	–	–	3.95	1.22
Dysfunctional attitudes - *Dependency*	3.30	1.09	–	–	3.68	1.22	3.52	1.18	–	–	3.50	1.32
Emotion regulation - *Suppression*	4.33	1.14	–	–	3.88	1.34	4.42	1.05	–	–	4.17	1.29
Emotion regulation - *Reappraisal*	4.01	1.05	–	–	4.09	1.15	4.02	1.09	–	–	3.87	1.14
Rumination - *Brooding*	2.86	0.66	–	–	2.56	0.74	2.73	0.63	–	–	2.72	0.70
Rumination - *Reflection*	2.41	0.59	–	–	2.08	0.64	2.40	0.65	–	–	2.32	0.74
Gender (% of girls)	91.0	–	–	–	–	–	83.2	–	–	–	–	–
Age	16.55	1.10	–	–	–	–	16.92	1.16	–	–	–	–

Ranges and anchors: Depressive Symptoms (0 = No symptoms, 60 = High level of and frequent symptoms); Negative automatic thoughts (1 = Not at all, 5 = All the time); Dysfunctional attitudes (1 = Strongly agree, 7 = Strongly disagree); Emotion regulation (1 = Strongly disagree, 7 = Strongly agree); Rumination (1 = Almost never, 4 = Almost always)

### ITT analyses

We used autoregressive latent variable models to assess the potential effects of the ACDC on depressive symptoms, negative automatic thoughts, dysfunctional attitudes, rumination, and emotion regulation. Pre-test variables that were associated with missingness were entered to the models either as control variables or as auxiliary variables. As outlined in the Appendix, these procedures contributed to a MAR assumption about missingness, justifying the application of the FIML as implemented in the Mplus program to account for missing data.

We used a dummy variable for condition to evaluate the intervention. For depressive symptoms, the latent variable of the post-test was regressed on the dummy controlling for the two pre-tests. For the other variables, we only had two time points, so the regression of the post scores on the dummy were controlled for the first pre-test scores only (in addition to controlling for gender and age, and also with auxiliary variables).

#### Primary outcome

##### Depressive symptoms

**Fig. 2:**
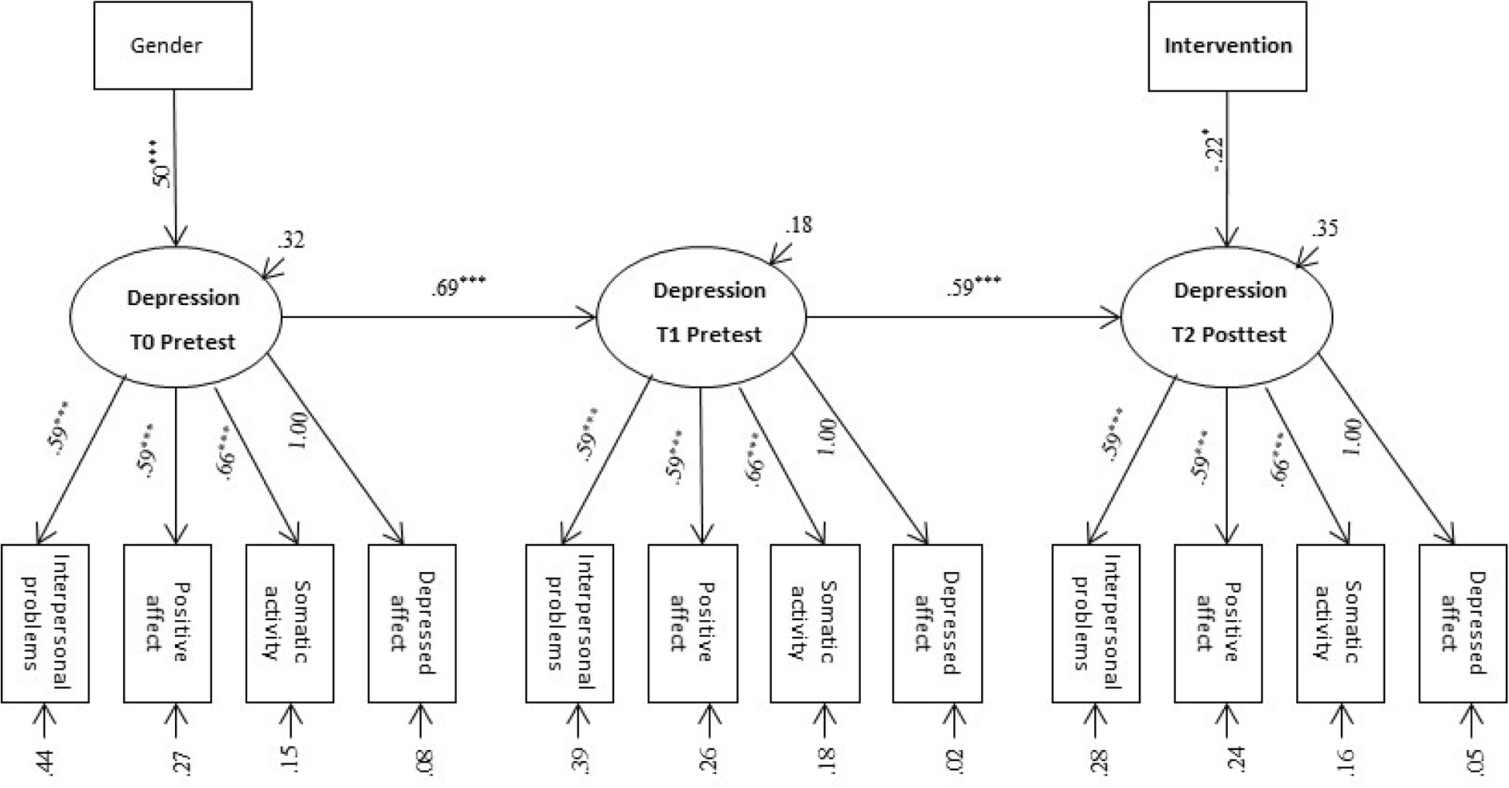
Path diagram of the longitudinal effect of the intervention on depression at post-test. Gender = Males are coded as 1 and females as 2. Intervention = UC control was coded as 0 and. ACDC intervention as 1. Unstandardized parameter estimates are reported only for the . significant paths from the covariates. *p < .05, ***p < .001

The latent variable model for depressive symptoms was estimated using the four sub-scales (depressed affect, lack of positive affect, somatic and retarded activity, interpersonal problems) as observed indicators. The residuals of identical indicators were correlated across time in accordance with SEM procedures for longitudinal measurements. Gender, age and condition of the intervention were entered as covariates, giving a mimic model approach. There were no significant associations between condition and depressive symptoms at T1 and T2. pre-test. A longitudinal invariant model gave close fit to the data, SRMR = .074; RMSEA = .040, 90% CI [.014, .059]; CFI = .98; TLI = .97. As can be seen from Fig. 2, the latent variable for the T3 score was regressed on the dummy variable for the intervention, controlling for the pre-test score and gender. There was a low standardized mean difference (d = −.31, *p* = .045) in favor of ACDC. Gender had a significant positive effect on the T1 score, indicating that girls reported higher levels of depressive symptoms at baseline.

#### Potential mediators/moderators

##### Negative automatic thoughts

The measurement model was assessed with eight items that gave acceptable fit to the data, SRMR = .070; RMSEA = .061, 90% CI [.049, .073]; CFI = .94; TLI = .93. As mentioned above, we only have the first pre-test (T1) and the post-test (T3) for all variables except for depression where we have all three points. The latent variable for the post-test was regressed on the dummy variable for condition, controlling for pre-test score as well as gender. Even though the standardized mean difference was in favor of the ACDC, the parameter was very low and not significant (d = −.09, *p* = .523).

##### Dysfunctional attitudes

The intensity of dysfunctional attitudes consists of the two dimensions “dependency” and “perfectionism/performance evaluation” with 6 and 11 items respectively. The two-factor solution gave acceptable fit to the data after removing one item for dependency due to low factor loading, SRMR = .083; RMSEA = .051, 90% CI [.044, .057]; CFI = .89; TLI = .88. There was an effect in favor of ACDC on perfectionism/performance evaluation (d = .34, *p* = .011). The parameter for the effect on dependency was in the same direction but not significant (d = .24, *p* = .114).

##### Emotion regulation

The measurement model assessed the two common emotion regulation strategies: cognitive reappraisal and expressive suppression, and the model gave an acceptable fit to the data, SRMR = .072; RMSEA = .040, 90% CI [.027, .051]; CFI = .94; TLI = .93. There were no significant effects of the intervention on these two variables.

##### Rumination

The two aspects of rumination (“reflection” and “brooding”) were measured by five items each, and the two-factor solution gave a good fit to the data, SRMR = .071; RMSEA = .044, 90% CI [.032, .055]; CFI = .93; TLI = .91. While the effect of the intervention variable was significant for reflection (d = −.35, *p* = .044), this was not the case for brooding (d = −.21, *p* = .186).

**Fig. 3:**
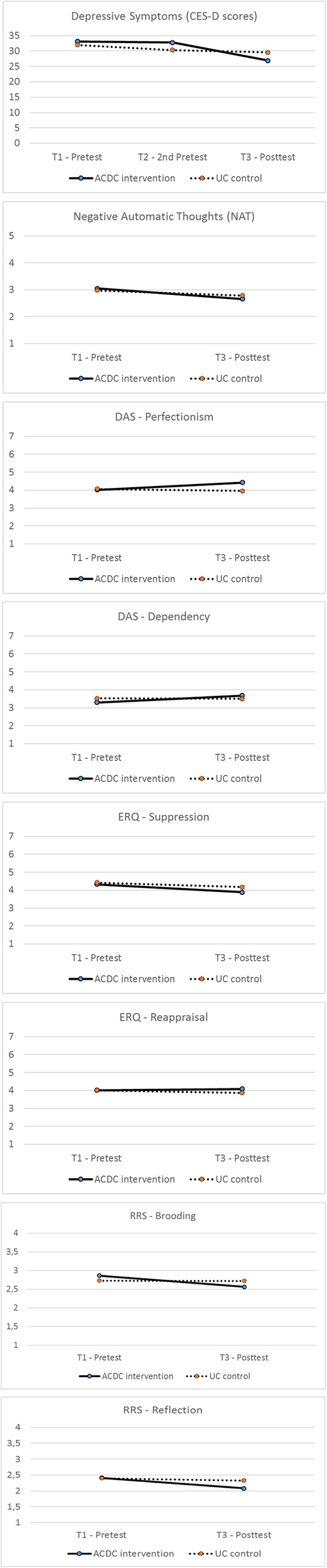
Slopes of improvement in the ACDC and UC groups

Table 2 provides the summary of the model fit statistics for the auto-regressive latent variable models for primary outcome measures and Table 3 shows the effects of the ACDC intervention for primary outcome measures for the ITT sample. See Fig. 3 for slopes of improvement in the ACDC and UC groups for each outcome.

**Table 2:** Model Fit Statistics for the Auto-regressive Latent Variable ITT Models

	χ^2^ Value	χ^2^ df	CFI	TLI	RMSEA	SRMR
Depression	92.45^*^	68	.978	.971	.040	.074
Negative automatic thoughts	244.81^***^	132	.936	.926	.061	.070
Dysfunctional attitudes	809.51^***^	511	.892	.881	.051	.083
Emotion regulation	270.85^***^	199	.937	.927	.040	.072
Rumination	285.37^***^	198	.925	.912	.044	.071

_*χ2*_ chi-square, *df* degrees of freedom, *CFI* the comparative fit index, *TLI* Tucker Lewis index, *RMSEA* the root-mean-square error of approximation, *SRMR* the standardized root-mean-square residual

^*^p < .05, ^***^p < .001

**Table 3:** Effects of the ACDC intervention

Outcomes	ITT *N* = 228		Sensitivity Analyses			
			ITT MNAR *N* = 228		Per Protocol *N* = 125	
	E.S.	*p*-value	E.S.	*p*-value	E.S.	*p*-value
Depression	−.31	.045	−.35	.019	−.35	.025
Negative automatic thoughts	−.09	.523	−.10	.476	−.09	.563
Dysfunctional attitudes - *Perfectionism/Performance evaluation*	.34	.011	.38	.006	.30	.081
Dysfunctional attitudes - *Dependency*	.24	.114	.31	.138	.09	.602
Emotion regulation - *Suppression*	−.11	.491	−.14	.537	.04	.834
Emotion regulation - *Reappraisal*	.21	.203	.20	.195	.37	.047
Rumination - *Brooding*	−.21	.186	−.23	.147	−.13	.239
Rumination - *Reflection*	−.35	.044	−.38	.025	−.16	.171

Standardized estimates (STDY) were presented

*ITT* Intention-to-treat analyses with the auxiliary variables, *ITT MNAR* Intention-to-treat analyses without the auxiliary variables, *E.S* Effect size

### Sensitivity analyses

#### Per protocol analyses

The sample used in the per protocol analyses (*N* = 125) consisted of those who did not violate the inclusion criteria and had data on all three waves. The reasons for exclusions were: 1) crossovers; participants initially randomized to ACDC but who received UC (*N* = 11), 2) falsely included; participants included in the study but subsequently found to be ineligible for reasons such as age > 20, lower level of BDI (*N* = 14), and 3) broken randomization; those who were informed about the condition they would receive prior to the first pre-test (*N* = 6). In addition, the 72 participants who did not have second pre-test and/or post-test data were excluded.

As the sample for the per protocol analyses consisted of 125 subjects only, the application of a latent variable approach could give biased results because there are too many parameters for this sample size. We, therefore, chose to report analyses based on sum scores. It must be kept in mind, though, that the application of sum scores may be biased by measurement errors. The per protocol analyses confirmed the results from our ITT analyses, although some of the effects were marginally significant. There was also a significant effect on cognitive reappraisal that was not significant for the ITT sample (see Table 3).

#### ITT MNAR analyses

As we have outlined above, our missingness was considered random (MAR) after entering several auxiliary variables that were associated with outcomes and their missingness. This means that removing the auxiliary variables from the models could result in a «missing not at random» situation (MNAR). A recommended procedure for sensitivity analyses is to re-analyse the data under the MNAR condition to see how sensitive the results are according to the missingness in the data. As can be seen from Table 3, the MNAR analyses without the auxiliary information inflated the significant effects a little compared to the ITT analyses that were conducted under the MAR assumption. Although none of these differences were significant, it underscores the importance of proper handling of missing data when analyzing data. Still, however, we can conclude that the analyses without the auxiliary information gave the same effects as the ITT analyses. Taken together with our per protocol analyses, this indicates that our findings are satisfactorily robust according to missingness.

### What kind of help did the UC control group receive?

The UC leaders reported from whom the adolescents received treatment. From the 95 participants that were allocated to UC, 16 of them (16.8%) got help from a psychologist. Four adolescents (4.2%) were referred to their GP (medical doctor), while 25 (26.3%) had conversations with the school nurse. Twenty (21.1%) adolescents had conversations with a teacher/educational counselor/other school personnel, while five (5.3%) were referred to child and adolescent psychiatric clinics. Three (3.2%) saw a physiotherapist and three (3.2%) had conversations with a clinical pedagogue. Five of the adolescents (5.3%) did not want any treatment or support, and for 13 (13.7%) of the adolescents we had no information We do not have information on whether any of the adolescents in the UC control group received evidence-based treatments for depression. However, if this occurred it was most likely to have been among those seeing a psychologist, clinical pedagogue, GP, or those referred to the psychiatric clinic (in total 29.5%). The remaining 70.5% most probably did not receive any evidence-based treatments.

### ACDC and UC leaders’ retrospective evaluations

We also asked the ACDC leaders to retrospectively evaluate whether they perceived the course as effective for the adolescents. We received valid answers on behalf of 112 of the young people. For 12 of the adolescents (10.7%) the course leaders reported that «No, it did not help». For 59 of the adolescents (52.7%) the course leaders reported that «Yes, it helped to a certain degree», while for 20 (17.9%) they reported «Yes, it helped a lot». For 21 adolescents (18.8%) the course leaders reported «Do not know». A fifth category «No, things got worse» was not ticked. As can be seen above, the median reported category was «Yes, it helped to a certain degree».

The leaders of the UC condition were also asked the same question regarding the help that their adolescents were given. We received answers on behalf of 104 adolescents. For one person it was «No, things got worse». For six individuals (5.8%) it was «No, it did not help». For 34 individuals (32.7% it was «Yes, it helped to a certain degree», and for 21 people (20.2%) it was «Yes, it helped a lot ». For 42 individuals the answer was «Do not know». Also for this condition, the median reported category was «Yes, it helped to a certain degree».

### Medication

In the post-test, the participants were also asked whether they used medication related to their depressive symptoms at the time of the data collection. Six individuals (8.2%) in the ACDC sample confirmed the use of medication, while two (2.7%) said that they did not know. For the UC sample, eight participants (11.9%) confirmed that they used medication, while two (3%) said that they did not know. This means that the use of medication was about the same in the two groups. When those who said they used medication were asked what kind of medications they used, four of the six using medication in the ACDC group answered anti-depressives, one answered anxiolytic and one answered “both”. For the UC group, four of the eight individuals that confirmed their use of medication answered anti-depressives while two answered both anti-depressives and anxiolytics.

### Course leaders’ self-reported fidelity

Four of the mean values for the intervention course leaders’ self-reported coverage of core treatment components were high. On the scale ranging from one to four, the ratings were *emotion regulation* (mean = 3.44, sd = .49), *the abc model (understanding own reactions/emotions)* (mean = 3.92, sd = .27), *significance of own thoughts* (mean = 3.73, sd = .45), *challenging own thoughts* (mean = 3.29, sd = .71). Two of the mean values were below three. The lowest rating was for *training tasks at home* (mean = 2.54, sd = .72), followed by *strengthening social relations* (mean = 2.93, sd = .80).

### Dosage

On average the adolescents receiving the intervention attended 6.5 of the 10 sessions with a standard deviation of 2.7.

## Discussion

The aim of this study was to evaluate the effectiveness of the Adolescent Coping with Depression Course (ACDC), a group cognitive-behavioral program for depressed adolescents aged 14–20 years, with subclinical, mild or moderate depressive symptoms. The active control group received “usual care” (UC) as implemented at the different sites. Adolescents receiving ACDC showed significantly lower depression scores at post-test compared to the UC group after controlling for the pre-test levels of depression. The effect size was small to medium (d = −.31). As expected, our effect size was in accordance with the one from the meta-analysis of Keles and Idsoe [30] (.28), Weisz et al. [31] (.34) and to the meta-analysis of the “Coping with Depression” course distributed to adolescents (.35) [32]. The 6-point reduction on the CES-D score for the intervention group goes from about 33 at pre-test to slightly below 27 at post-test, indicating that the average score is below the cutoff suggested by Manson et al. [43]. We suggest this as a clinically meaningful symptoms reduction. The same directions of effects were found for all the other variables, however, only two out of seven effects were significant.

Negative automatic thoughts are considered important when it comes to depression. These quick evaluative thoughts that individuals are barely aware of are expected to be associated with depression, and a core issue within CBT is to identify and modify these thoughts for depression recovery. Even though the effect size was in the expected direction, it was lower than expected, based on the attention it receives within CBT and the ACDC. Automatic thoughts are hypothesized to arise from more stable cognitions and coping styles, and dysfunctional attitudes like “dependency” and “perfectionism/performance evaluation” are one kind of such cognitive vulnerabilities. Both of these attitudes demonstrated effects in the expected direction, however, only the one for perfectionism/performance evaluation was significant. This may indicate that the intervention was better tailored to deal with perfectionism/performance evaluation. But it could also be that dependency is not so easy to change. The same occurred for the two aspects of rumination; that is the intervention had a significant effect on reflection but not on brooding. For the two emotion regulation variables, we found no significant effects. Even though small sample size could be the reason for the non-significant effects, the parameters were still low. Taken together this means that the manual for ACDC could be inspected to see whether there are any potential reasons why some of the expected effects did not reach significance. Another reason may be that the duration, dosage and intensity of the interventions was insufficient to promote the necessary change in thoughts and feelings. The group format may also have been difficult for some of the participants, and some may have needed individual help and support.

No matter what, the associations between depression and the cognitions referred to above should be investigated within a longitudinal design to inspect possible directions of effects. This will be possible in our study when information from more time points are gathered.

The ACDC and UC leaders also gave their retrospective evaluations of whether they perceived that the treatments helped the adolescents. For both conditions, most adolescents were assumed to have benefitted from the help they received to a certain degree. Although the ACDC leaders reported that the treatment did not help for about 10 % of the adolescents compared to only about 5 % of the UC sample, more UC leaders reported that they did not know whether the treatment helped, compared to the ACDC leaders. So even though the retrospective evaluations provided by the ACDC/UC leaders did not discriminate the perceived effect of the two conditions, this was definitely established within our effect analyses.

The ACDC leaders’ self-reports demonstrated high adherence to core treatment components. However, future studies should investigate this within a better design by providing observation data.

There were a number of limitations to this study. First of all, even though the effect on depression was within the expected range and significance, the sample size was probably too small to detect some of the effects for the other outcomes. Future studies should investigate the same research questions with larger sample sizes. In addition, as the RCT was conducted in a highly natural setting, as effectiveness researchers, we had limited control over factors such as screening and inclusion of participants, and appropriate implementation of the intervention. The challenges with recruitment of participants and recruiting fewer boys may also indicate that it may be difficult for adolescents to admit that they have problems they may need help for, and this may apply particularly for boys. The fear of being singled out as depressed or as a ‘mental health case’ may have stopped some students volunteering to attend. These factors may affect our interpretation of the results of this RCT. Depression symptoms like sadness, lack of energy and low self-esteem reduce motivation for seeking help. More proactive recruiting procedures should be considered. The use of an active control may have contributed to the relatively modest effect size observed. It is likely the effect size would have been somewhat larger if a wait-list control group was applied. Adolescents with ADHD or those struggling with substance use do not satisfy the inclusion criteria of the intervention, so we cannot generalize our findings to such subgroups. The fidelity is measured by self-report only and may very well be skewed. Future studies should investigate this by use of observations. Another issue is that as long as the amount of contact in the treatment group was higher than for the control group, this may have accounted for some of the positive results regardless of the content of the treatment. Future studies should more strictly disentangle a potential attention effect from a potential treatment effect. Furthermore, as approximately 85% of participants in the study were female, this raises a question as to whether the results can be generalized to depressed males. Our control for gender should therefore be carefully interpreted. Another limitation is that we only rely on self-report measures. Participants also were not blind to their treatment condition after the interventions started. This may affect their expectations, that could again bias their ratings. Having an independent evaluator who is blind to treatment arm, and including parental reports, would have strengthened the study. The self-report measure for depression was administered at T1, T2 and T3. This made it possible to control for potential clinical improvement after T1 but prior to treatment. We considered this important, as depression was our primary outcome. However, our remaining study variables were only assessed at T1 and T3, limiting our possibilities to control for potential clinical improvement prior to treatment for these study variables. Even though our analyses at the individual level may be statistically justified because of low design effects (less than 2), this does not mean that course leaders are not important. Our study was not powered to detect potential effects at the cluster level (for the course leaders). Future studies should increase the number of clusters. Another limitation is the lack of data on homework completion. Future research should investigate whether homework completion is associated with improvement among subclinical and mildly depressed youth, or if there are variations in homework completion depending on severity.

There is a loss of statistical power resulting from the attrition rates for the per protocol analyses, but we argue that the attrition did not bias our estimates. We investigated the missingness thoroughly and found no significant differences between the attrition group and those who continued for post-test. Furthermore, we contacted the attrition group and asked their reasons for not continuing. Most of these reasons were practical issues, and we found no specific differences in reported reasons across conditions. Finally, we added auxiliary information to our ITT analyses, supporting our assumption of a MAR mechanism behind missingness. In addition to including such predictors of missingness, we carried out sensitivity analyses (per protocol analysis and MNAR analysis). Our ITT analyses based on the FIML approach was mainly supported by the sensitivity analyses. This strengthens our findings. The longitudinal invariance of our measures also supports the assumption that we have measured the same constructs at all time points [60].

As seen also in our case, studies have demonstrated that the levels of fidelity do not reach 100% in the implementation of various programs [61]. Still, one of the strengths for the practice field that warrants mentioning is the ease with which numerous clinicians of various backgrounds were trained to administer this evidence-based protocol with (self-reported) fidelity. It should also be kept in mind that some of our course facilitators were newly trained, but still our results show ACDC is effective in the real life setting despite the variation in their level of experience.

Lastly, even though RCTs have been considered as the ‘gold standard’, they are also criticized as lacking ‘external validity’ [62]. Hence, using additional qualitative data with the aim of capturing more contextual knowledge and participants’ own perspectives may benefit future RCTs [62].

## Conclusion and implications

Based on ITT analyses supported by sensitivity analyses, our study provides support for the effectiveness of the group-based CBT intervention course ACDC. This can hopefully result in clinically significant reductions in symptoms associated with depression among adolescents. However, our difficulties related to recruitment and attrition raise important issues which need to be solved in order to increase the possibility of these treatment programs to be disseminated. Treatment is largely only offered when children and adolescents ask for it. However, research into help seeking behavior shows that less than 20% of young people in need seek help with their problems [15]. More proactive strategies are needed to reach those who need help and to implement these programs in an effective way. Effective, more systemic and structured identification and recruitment routines for adolescents with mental health problems are probably crucial, as is better cooperation between different services involved with adolescents in need. Future studies with a cross-over design, with UC followed by enrolment in ACDC, could be interesting in addressing the recruitment challenge. A potential module in the treatment focusing on therapeutic rapport and alliance could also be implemented to see whether this improved attendance and outcome.

To conclude, the ACDC can be delivered by a broad array of providers, trained in a relatively short period of time and result in reductions in self-reported depression in adolescents. This is a contribution to the literature, and hopefully a useful intervention for the practice field.

## Appendix

### Attrition Analyses

The comparison analyses (chi-square analyses for gender and t-test analyses for age and each outcome measure) for the participants dropped out versus those remained in following measurements are presented in the following table, based on their information at T1-pre-test.

**Table:**

	Participating T2-2nd pre-test vs. not		Participating T3-post-test vs. not	
	*t-test/*χ^2^	*p* value	*t-test/*χ^2^	*p* value
*Demographic Characteristics*				
Gender	1.807	.179	.303	.582
Age	3.061	.002	.191	.849
*Outcomes*				
Depression T1-1st pre-test	.731	.465	1.081	.281
Depression T2-2nd pre-test	–	–	−.091	.928
Negative automatic thoughts	−.223	.824	.106	.916
Dysfunctional attitudes	−.073	.941	−.569	.570
Emotion regulation	1.844	.066	1.636	.103
Rumination	1.200	.232	1.352	.178

### Details Regarding Statistical Analyses and Missing Data

SEM was preferred because of several strengths such as: 1) enabling the use of multiple observed indicators of each latent variable and thereby reducing bias due to errors of measurement, 2) adjusting for potential bias in estimates of standard errors due to cluster randomization, 3) modeling more complex variance and covariance structures across time, 4) being able to estimate indirect effects, and 5) enabling us to include cases with partly missing data. We used a dummy variable to represent the two groups. The UC group was used as the reference group and coded as zero. Effect sizes (d values) for the intervention were calculated based on the partial regression coefficients for the dummy variable as reported in the STDY table of the Mplus output.

In order to apply the FIML procedure, missingness must be considered completely at random - MCAR (the same as for being able to run listwise deletion) or the less restrictive «missing at random» assumption - MAR, meaning that the missingness is considered random after controlling for measured variables that are related to the missingness. In order to examine the missing mechanism, and to deal with missingness in an appropriate manner, we took several steps. First, we examined the participant flow in detail, and whether study dropout was different by condition. Then, we contacted subjects who dropped out to get information about the reasons for dropout. The responses from the attrition group did not reveal any systematic differences in the reasons for dropping out by the condition. Moreover, many of the reasons reported were in relation to the practical and/or administrative issues, and the methodological literature suggests that such reasons for missingness are very often random [58]. Last but not least, the FIML approach can also account for factors leading to missingness by use of auxiliary information that is relevant for the dropouts [57]. The information gathered in the first pre-test was used for this purpose. This means that all of our ITT analyses have auxiliary variables included. In sum, we assumed missingness to be random (MAR) after investigating the missing data patterns, inspecting the answers from the dropouts, and after controlling for the auxiliary information that we added to the models.

### Overview of the training protocol for ACDC course leaders

1. Part A
  - Target group
  - Recruitment
  - Preparatory conversation
  - Evaluation
  - Evaluation of participation
2. Part B Methods and theory
3. Part C Implementation of the course
  - How feelings arise
  - Situations and thoughts that can lead to sadness
  - Regulating feelings with thoughts
  - Regulating feelings with actions
  - Thinking in better ways
  - Training in unaccustomed thoughts: Positive thinking and contact
  - Better contact with others
  - Coping and cognitive techniques
  - Daily use of methods. Work and practice
  - Daily use of methods. Conclusion
4. Part D
  - Facilitation
5. Part E
  - Tools
  - Some signs of depression
  - ICD-10 symptoms of depression
  - Cognitive style and cognitive thinking errors
  - Optional mapping at the course
  - Some internet resources

## Abbreviations

ACDC: The Adolescent Coping with Depression Course
UC: Usual care
